# Effects of Vitamin B6 and Folic Acid on Cardiometabolic Biomarkers and Cardiac Oxidative Stress in Homocysteine-Loaded Rats

**DOI:** 10.3390/biomedicines14061373

**Published:** 2026-06-18

**Authors:** Dušan Todorović, Marija Stojanović, Slavica Mutavdžin Krneta, Jovana Jakovljević Uzelac, Nina Radisavljević, Kristina Gopčević, Ana Medić, Milica Labudović Borović, Jelena Rakočević, Sanja Stanković, Dragan Djuric

**Affiliations:** 1 Institute of Medical Physiology “Richard Burian”, Faculty of Medicine, University of Belgrade, 11000 Belgrade, Serbia; mrj.stojanovic@gmail.com (M.S.); slavica.mutavdzin@gmail.com (S.M.K.); jovanavjakovljevic@gmail.com (J.J.U.); nina_radisavljevic@outlook.com (N.R.); dr_djuric@yahoo.com (D.D.); 2 Institute of Chemistry in Medicine “Petar Matavulj”, Faculty of Medicine, University of Belgrade, 11000 Belgrade, Serbia; kristinagopcevic@yahoo.com (K.G.); medicana89@gmail.com (A.M.); 3 Institute of Histology and Embryology “Aleksandar Ð. Kostić”, Faculty of Medicine, University of Belgrade, 11000 Belgrade, Serbia; sborovic2001@yahoo.com (M.L.B.); koskabg@gmail.com (J.R.); 4 Center for Medical Biochemistry, University Clinical Center of Serbia, 11000 Belgrade, Serbia; sanjast2013@gmail.com; 5 Faculty of Medical Sciences, University of Kragujevac, 34000 Kragujevac, Serbia

**Keywords:** aorta, folic acid, heart, homocysteine, hyperhomocysteinemia, rat, vitamin B6

## Abstract

**Background/Objectives:** Elevated homocysteine (Hcy) levels are associated with endothelial dysfunction, oxidative stress, and inflammation, contributing to cardiovascular disease development. The aim of this study was to examine the effects of vitamin B6 and folic acid on cardiometabolic biomarkers, cardiac oxidative stress, metabolic enzyme activities and cardiovascular histomorphometric parameters in homocysteine-loaded rats. **Methods:** Male *Wistar albino* rats were divided into four groups (n = 10, per group): C—saline 0.2 mL/day s.c. + saline 0.5 mL i.p; H: Hcy 0.45 µmol/g b.w./day s.c. + saline 0.5 mL i.p; C-B6+FA—saline 0.2 mL/day s.c. + vitamin B6 (7 mg/kg b.w. i.p./day) and folic acid (FA) (5 mg/kg b.w. i.p./day); and H-B6+FA—Hcy (0.45 µmol/g b.w./day s.c.) + vitamin B6 (7 mg/kg b.w. i.p./day) and FA (5 mg/kg b.w. i.p./day). Substances were applied s.c. for 2 weeks and i.p for 4 weeks. **Results:** B6+FA supplementation significantly reduced serum Hcy and LDL concentrations and attenuated Hcy-associated increases in cardiac SOD activity and right ventricular wall thickness. However, B6+FA was associated with increased cardiac MDA levels. MDH activity increased mainly in non-Hcy-loaded supplemented rats, whereas LDH activity, the cardio-somatic index, and aortic histomorphometric parameters remained unchanged. **Conclusions:** Combined B6+FA supplementation may improve Hcy metabolism, the LDL profile, and selected cardiac structural/oxidative alterations, but its association with increased lipid peroxidation suggests complex redox effects requiring cautious interpretation.

## 1. Introduction

Sulfur-containing amino acids (SAAs) are a group of amino acids and related metabolites that contain a sulfur atom in their side chain, giving them unique biochemical roles. The main SAAs in humans are methionine (Met) and cysteine (Cys), which provide sulfur for protein synthesis and are central to one-carbon metabolism and redox balance [1]. SAAs enter the body primarily in the form of Met and Cys, which are present in dietary proteins [2]. Met is an essential amino acid and precursor to S-adenosylmethionine, while Cys, formed via trans-sulfuration, is a key source of glutathione and other sulfur metabolites like taurine. Hcy, a non-proteinogenic SAA derived from Met, acts as a critical intermediate, with plasma levels linked to cardiometabolic outcomes [3]. Altered plasma concentrations of Met, Cys, Hcy, cystathionine, glutathione, cysteine sulfinic acid and taurine are associated with obesity and cardiometabolic risk, highlighting their systemic importance in health and disease [1]. The reference range for blood Hcy levels in healthy individuals is typically between 5 and 15 µmol/L. Hyperhomocysteinemia (HHcy) is defined as a clinical state characterized by a sustained elevation of plasma Hcy concentration exceeding 15 µmol/L [4].

HHcy may contribute to the development of cardiovascular diseases, including ischemic heart disease and myocardial infarction, cerebrovascular disorders (ischemic stroke), peripheral vascular disease and thrombotic conditions, neurodegenerative and cognitive impairments (dementia and neurological disorders), osteoporosis and an elevated risk of fractures, and the progression of chronic kidney disease [5,6,7].

Recent experimental studies in rodent models have demonstrated that HHcy is associated with significant impairment of key antioxidant defenses, including reductions in the activities of SOD and catalase (CAT) within cardiac tissue, reflecting a diminished capacity to neutralize reactive oxygen species (ROS) and protect cellular structures from oxidative damage [8]. Increased oxidative stress under conditions of elevated Hcy is further evidenced by enhanced lipid peroxidation, as indicated by raised levels of MDA, a widely used marker of oxidative injury to membrane lipids that correlates with cardiovascular risk and disease progression [8].

Hyperhomocysteinemia also perturbs lipid metabolism and lipid profiles, contributing to an atherogenic milieu characterized by dysregulated cholesterol and lipoprotein dynamics that are known to exacerbate endothelial dysfunction and promote atherosclerotic plaque formation. Although specific clinical correlations vary, observational studies have linked higher Hcy levels with adverse lipid indices and reduced antioxidant capacity in patients with ischemic heart disease [9]. At the cellular level, oxidative imbalance triggered by Hcy not only undermines enzymatic antioxidant protection but also influences the activities of key metabolic enzymes involved in energy metabolism. For example, alterations in cardiac lactate dehydrogenase (LDH) and MDH activities have been observed in models of cardiometabolic stress, suggesting that Hcy-associated oxidative conditions modulate enzymatic pathways that are central to myocardial substrate utilization and redox homeostasis [10].

The literature provides extensive evidence that Hcy exerts detrimental effects on the endothelium by disrupting its physiological functions. One of the pathophysiological impacts of Hcy on the endothelium is the impairment of its ability to regulate vascular tone. Endothelial cells maintain vascular tone through the release of various mediators, such as nitric oxide (NO), prostacyclin, endothelin-1 (ET-1), and thromboxane A_2_ (TXA_2_) [11]. Studies have demonstrated that Hcy reduces the bioavailability of vasodilator NO, whereas circulating Hcy levels show a positive correlation with concentration of ET-1, a potent vasoconstrictor, in individuals with impaired glucose metabolism and vasculitis [12]. Enhanced biosynthesis of TXA_2_ has also been observed in patients with elevated plasma Hcy levels [13]. Either directly or through increased production of ROS, Hcy is associated with the initiation of inflammatory processes within vascular tissues, further contributing to the progression of endothelial dysfunction [14]. Cell-culture studies have demonstrated that Hcy induces the production of pro-inflammatory cytokines such as monocyte chemoattractant protein-1 and interleukin (IL)-8 via activation of the nuclear factor kappa B (NF-κB) signaling pathway [15]. NF-κB is a transcription factor that promotes the expression of cytokines, chemokines, and adhesion molecules, thereby contributing to altered endothelial cell function and leukocyte recruitment, which collectively accelerate the development of atherogenesis. Studies conducted in apolipoprotein E-deficient mice with induced HHcy have likewise demonstrated activation of the NF-κB signaling pathway and elevated levels of pro-inflammatory mediators within atherosclerotic lesions [16,17].

Hcy derived from Met can follow several distinct metabolic fates: it may be remethylated back to Met by enzyme methionine synthase (MS) (this metabolic route is referred to as the “remethylation cycle” because the Hcy molecule regains the methyl group that was removed from S-adenosylmethionine through the action of methyltransferases in preceding reactions); it may enter the trans-sulfuration pathway, in which it is ultimately converted to Cys; it may be transformed into toxic metabolite Hcy-thiolactone through the action of methionyl-tRNA synthetase; or it may form a disulfide bond with the side chain of a Cys residue incorporated into a protein’s polypeptide chain [18,19,20].

Enzyme MS uses 5-methyltetrahydrofolate (THF-CH_3_) as a substrate from which it transfers a methyl group for the remethylation of Hcy. Vitamin B_12_ serves as the cofactor for MS. THF-CH_3_ is a derivative of folic acid (FA) [21]. In the trans-sulfuration pathway, Hcy is converted to Cys through two reactions. The first reaction involves the condensation of Hcy and serine, catalyzed by enzyme cystathionine β-synthase (CBS), resulting in the formation of cystathionine. In the second reaction, cystathionine γ-lyase (CGL) catalyzes the breakdown of cystathionine into α-ketobutyrate and Cys. Both CBS and CGL enzymes require vitamin B_6_ as their cofactor [21]. Vitamin B6 and FA have important but distinct roles in Hcy metabolism and HHcy-related pathophysiology. In experimental models, FA supplementation has been shown to reduce methionine-induced increases in plasma Hcy in hyperhomocysteinemic rats, supporting the importance of folate-dependent remethylation in Hcy clearance [22]. In addition, vitamin B6 status appears to influence the oxidative consequences of elevated Hcy, as vitamin B6 deficiency was found to aggravate homocysteine-induced oxidative stress and alter glutathione-related antioxidant responses in mice [23]. In humans, meta-analytic evidence from randomized trials indicates that folic acid supplementation significantly lowers circulating Hcy concentrations, whereas vitamin B6 alone has a less consistent effect on fasting Hcy levels, suggesting that combined B-vitamin approaches may have context-dependent metabolic and cardiovascular relevance [24].

According to its underlying etiology, HHcy can be classified into two categories. The first category comprises rare but severe forms of HHcy caused by major genetic mutations affecting enzymes responsible for Hcy metabolism. The second category includes milder yet more common forms characterized by only modest elevations in plasma Hcy levels that arise from a combination of environmental factors and minor genetic variants [25,26].

Given the clinical relevance of HHcy to numerous pathophysiological processes within the cardiovascular system, as well as the importance of vitamin B_6_ and FA in its metabolism, we hypothesized that experimental Hcy loading would induce adverse cardiometabolic, oxidative, metabolic, and structural alterations in the cardiovascular system. Specifically, we expected Hcy loading to increase circulating Hcy levels, disturb serum lipid and hemostatic biomarkers, enhance oxidative stress in cardiac tissue, alter cardiac metabolic enzyme activities, and promote histomorphometric changes in the heart and aortic wall. Furthermore, we hypothesized that combined vitamin B6 and folic acid supplementation would exert a protective effect by lowering serum Hcy concentrations and attenuating Hcy-induced biochemical and structural alterations. Finally, using a 2 × 2 factorial design, we aimed to determine not only the independent effects of Hcy loading and vitamin supplementation but also whether vitamin B6 and folic acid modify the cardiovascular effects of Hcy, as reflected by the Hcy × B6+FA interaction. The contribution of the present study lies in its comprehensive evaluation of combined vitamin B6 and folic acid supplementation in an experimental model of Hcy loading. Unlike studies that focus only on circulating Hcy levels or individual oxidative stress markers, this study simultaneously assessed systemic cardiometabolic and hemostatic biomarkers, cardiac oxidative stress parameters, metabolic enzyme activities, and cardiovascular histomorphometric outcomes.

## 2. Materials and Methods

### 2.1. Experimental Animals

Male Wistar albino rats served as the experimental model. At the beginning of the study, animals were 15–20 days old and had an average body mass of approximately 160 g. They were supplied by the Vivarium of the Military Medical Academy in Belgrade. Housing was arranged in transparent plexiglass cages, accommodating two animals per cage, with sawdust used as bedding material. Animals had unrestricted access to standard laboratory feed and drinking water. Environmental parameters were carefully controlled throughout the experimental period. Room temperature was maintained at 21 ± 2 °C; relative humidity was maintained at 55 ± 5%; and illumination followed a 12 h light/dark schedule, with lights turned on at 07:30 h. Before the experimental protocol commenced, all animals underwent a three-day period of acclimation to laboratory housing conditions.

Ethical approval for the study was obtained from the Ethics Committee for the Welfare of Experimental Animals of the Ministry of Agriculture, Forestry and Water Management, Veterinary Directorate, Republic of Serbia (approval number 323-07-02523/2018-05), with formal authorization issued on 19 March 2019. All animal-related procedures were carried out in accordance with current animal welfare regulations and internationally recognized ethical standards, including the provisions of EU Directive 86/609/EEC governing the use of vertebrate animals for experimental and scientific purposes.

### 2.2. Experimental Protocol

**Figure 1:**
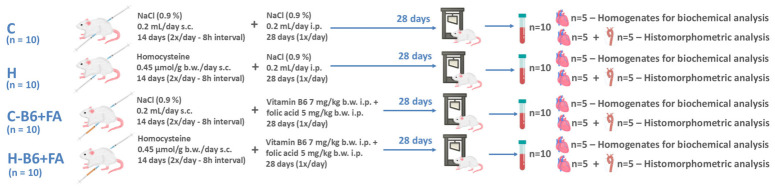
Schematic of the experimental pipeline. Male Wistar albino rats were divided into four groups, receiving saline, homocysteine, vitamins (B6 and folic acid), or a combination of homocysteine and vitamins according to the shown experimental regimen. After 28 days of treatment, animals were euthanized by decapitation, and blood samples were collected (n = 10 per group) for biochemical analyses in serum and plasma, while the heart tissue was allocated for biochemical (n = 5 per group) and histomorphometric (n = 5 per group) analyses and aortic tissue (n = 5 per group) was allocated for histomorphometric analyses.

The animals were divided into 4 groups and treated according to the following regimen: C group (n = 10)—administration of saline solution (0.9% NaCl, 0.2 mL s.c.) twice daily at 8 h intervals for 14 consecutive days and saline solution (0.9% NaCl, 0.5 mL i.p.) once daily for 28 consecutive days; H group (n = 10)—administration of D,L-Hcy (0.45 µmol/g b.w. s.c.) [27] twice daily at 8 h intervals for 14 consecutive days and saline solution (0.9% NaCl, 0.5 mL i.p.) once daily for 28 consecutive days; C-B6+FA group (n = 10)—administration of saline solution (0.9% NaCl, 0.2 mL s.c.) twice daily at 8 h intervals for 14 consecutive days and vitamin B6 (7 mg/kg b.w. i.p.) and FA (5 mg/kg b.w. i.p.) once daily for 28 consecutive days; and H-B6+FA group (n = 10)—administration of D,L-Hcy (0.45 µmol/g b.w. s.c.) twice daily at 8 h intervals for 14 consecutive days and vitamin B6 (7 mg/kg b.w. i.p.) and FA (5 mg/kg b.w. i.p.) once daily for 28 consecutive days (Figure 1). Hcy was administered s.c. during the first two weeks of the experimental protocol, whereas vitamin B6 and FA were administered i.p. throughout all four weeks of the protocol.

According to guidelines issued by the National Research Council Subcommittee on Laboratory Animal Nutrition, the estimated physiological requirement for FA in rats is approximately 2 mg/kg/day [28]. Diets supplemented with 8 mg/kg FA are classified as providing a moderate level of intake and are considered comparable to a daily folic acid consumption of approximately 1–6 mg in humans, which falls within recommended ranges for human supplementation [29]. Based on these considerations, a daily FA dose of 5 mg/kg was selected for use in the present study.

The recommended daily intake of vitamin B_6_ for rats, as defined by the same National Research Council subcommittee, ranges from 6 to 7 mg/kg [28]. In this experimental design, vitamin B_6_ was administered at the upper recommended level (7 mg/kg/day) in order to evaluate whether supplementation at a physiologically relevant dose exerts beneficial effects under normal conditions, as well as in animals with experimentally induced HHcy.

At the conclusion of the 28-day treatment period, animals were euthanized by decapitation using a small animal guillotine. Immediately thereafter, blood samples were collected (n = 10 per group), followed by surgical excision of the heart and aorta. Cardiac mass was determined in all animals (n = 10 per group), and the cardiac somatic index (CSI) was calculated as the ratio of heart weight (g) to total body weight (g). Out of ten animals per group, heart samples from five animals (labeled 1–5) were used for biochemical analysis, while heart samples from the remaining five animals (labeled 6–10) were used for histological and histomorphometric analysis. Histological and histomorphometric analysis of aortic tissue was also performed in five animals (labeled 6–10).

### 2.3. Determination of Biochemical Parameters in Serum and Plasma

Blood samples were obtained immediately after termination of the experiment and processed for biochemical evaluation. For plasma preparation, blood was collected into citrate-containing tubes, while samples intended for serum analysis were kept at ambient temperature to allow for natural clot formation before separation. Fractionation of plasma and serum was achieved by centrifugation at 3000 rpm for 15 min, and the resulting supernatants were subsequently used for analysis. The measured parameters included cardiovascular serum biomarkers—namely, the concentrations of Hcy, total cholesterol, LDL, high-density lipoprotein, triglycerides, high-sensitivity troponin T, vitamin B12, and folate, as well as LDH activity—and hemostatic plasma biomarkers—namely, fibrinogen, D-dimer, and vWF. The parameters were measured using an automated biochemical analyzer (Dimension Xpand, Siemens, Erlangen, Germany) via spectrophotometry or with commercial kits (Siemens Healthcare Diagnostics Ltd., Frimley, Camberley, UK).

### 2.4. Preparation of Heart-Tissue Homogenates for Biochemical Analysis

For biochemical measurements, cardiac tissue was first processed to obtain cell lysates. Samples were suspended in an ice-cold extraction medium composed of Tris-Cl (20 mmol/L, pH 7.5), sucrose (250 mmol/L), Triton X-100 (1%), phenylmethylsulfonyl fluoride (1 mmol/L), and leupeptin (1 µg/mL). Tissue was combined with buffer at a ratio of 1:10 (*w*/*v*) and mechanically disrupted on ice using an electric homogenizer (Witeg Labortechnik GmbH, Wertheim, Germany). The resulting suspension was clarified by centrifugation at 10,000 rpm for 10 min at 4 °C, and the obtained supernatant was collected as the total protein extract. Prepared samples were stored at −20 °C until further analysis. All biochemical endpoints in the heart lysates were quantified by spectrophotometric methods using a UV-2600 spectrophotometer (Shimadzu, Kyoto, Japan). Protein content in all tissue homogenates was determined using the Bradford assay. Briefly, 0.2 mL of each homogenate was combined with 1 mL of Bradford reagent (Sigma-Aldrich, Darmstadt, Germany) and allowed to react at room temperature for 15 min. Absorbance was then measured at 595 nm using a spectrophotometer. Distilled water served as a blank in place of the homogenate. Protein concentrations were calculated from a standard curve generated with known concentrations of bovine serum albumin.

### 2.5. Determination of Oxidative Stress Parameters in Heart-Tissue Homogenates

CAT activity in tissue homogenates was determined spectrophotometrically by tracking the decomposition of hydrogen peroxide at 260 nm [30]. The assay mixture consisted of 200 µL of homogenate, 1 mL of 0.18% H_2_O_2_ solution in 0.05 M phosphate buffer (pH 7.0), and 2 mL of the same buffer.

Total SOD activity in tissue homogenate samples was evaluated using a technique that measures the inhibition of epinephrine auto-oxidation to adrenochrome at 360 nm under alkaline conditions (pH 10.2) [31]. The reaction mixture contained 100 µL of homogenate, 1.8 mL of 50 mM Tris-Cl buffer (pH 10.2), and 0.1 mL of 0.18 g/L epinephrine solution prepared in 0.1 M HCl.

Lipid peroxidation in tissue homogenates was assessed by quantifying MDA using the thiobarbituric acid reactive substances method [32]. Briefly, 10 µL of homogenate was combined with 10 µL of 10% sodium dodecyl sulfate and 980 µL of a reagent composed of equal parts of 7 mM thiobarbituric acid in 20% acetic acid and 1 M NaOH. The mixture was heated in a boiling water bath at 100 °C for 30 min, then rapidly cooled on ice for 10 min. Following centrifugation at 4000 rpm for 5 min to remove precipitated proteins, the clear supernatant was transferred to cuvettes, and absorbance was recorded at 532 nm. The MDA concentration was calculated using the Lambert–Beer equation with a molar extinction coefficient of 1.56 × 10^5^ M^−1^·cm^−1^, expressed as µmol per mg of protein in the homogenate.

### 2.6. Determination of Metabolic Enzyme Activities in Heart-Tissue Homogenates

LDH activity in tissue homogenates was assessed spectrophotometrically by tracking the decrease in nicotinamide adenine dinucleotide + hydrogen (NADH) absorbance at 340 nm [33]. The reaction mixture consisted of 0.01 mL of homogenate, 2.9 mL of 0.1 M phosphate buffer (pH 7.0), 0.1 mL of 23 mM sodium pyruvate, and 0.05 mL of 14 mM NADH, all prepared in distilled water. Absorbance changes were recorded over the first 3 min at 25 °C.

MDH activity was measured similarly, monitoring NADH oxidation at 340 nm. [34]. The assay mixture contained 0.01 mL of homogenate, 2.9 mL of 0.1 M phosphate buffer (pH 7.5), 0.1 mL of 15 mM sodium oxaloacetate, and 0.05 mL of 14 mM NADH. The change in absorbance was followed for 3 min at 25 °C.

### 2.7. Histological and Histomorphometric Analysis of Heart and Aortic Tissues

For each experimental group, five heart and five aorta samples were selected for histological and morphometric evaluation. Tissue specimens were first immersed in 4% neutral buffered formaldehyde at room temperature for at least 24 h. After fixation, samples were dehydrated in a graded series of alcohols and cleared in xylene using an automated tissue processor. Paraffin embedding was performed with an embedding station, and blocks were trimmed before sectioning. Using a rotary microtome, 3 µm sections were obtained; transverse heart sections were taken at the mid-ventricular level. All sections were stained with hematoxylin and eosin/phloxine (H&E) to allow for both morphological and histomorphometric analyses.

Microscopic observations were made with an Olympus BX41 microscope (Tokyo, Japan) fitted with a wide-zoom camera, and images were analyzed using ImageJ software (https://imagej.net/ij/download.html; Access date: 12 July 2023). In the heart tissue, the presence of ischemic alterations and connective tissue proliferation was evaluated. Morphometric parameters included left and right ventricular wall thickness, interventricular septum thickness, and cardiomyocyte transverse diameter measured at the nuclear level. Each parameter was recorded ten times per heart section. In the aorta, assessment focused on atherosclerotic lesions and structural remodeling. Measurements included tunica media thickness, spacing between elastic laminae, and the total number of laminae, each measured ten times per aortic section.

### 2.8. Chemicals Used in the Study

Hcy for the induction of HHcy was obtained from Sigma-Aldrich Chemie GmbH (Schnelldorf, Germany). For the treatment of rats, vitamin B6 (pyridoxine) (Sigma-Aldrich Corporation, St. Louis, MO, USA) and FA (Sigma-Aldrich Corporation, St. Louis, USA) were used. Chemicals for the quantification of biochemical parameters were procured from Sigma-Aldrich Corporation (St. Louis, USA). Laboratory consumables were obtained from Eppendorf (Hamburg, Germany).

### 2.9. Statistical Analysis

All statistical analyses were performed using SPSS software (version 23.0), while graphs were generated using GraphPad Prism software (version 9.0.0). Data distribution was assessed using the Shapiro–Wilk test, and homogeneity of variances was evaluated using Levene’s test. The effects of Hcy loading and combined vitamin B6 and folic acid supplementation were analyzed using a two-way general linear model (two-way ANOVA), with Hcy and B6+FA as fixed factors, including their interaction term (Hcy × B6+FA). When the assumptions of normality and/or homogeneity of variance were not fulfilled, data were log10-transformed before analysis, and the transformed values were subsequently analyzed using the same two-way general linear model. Results are presented as means ± standard deviations for normally distributed variables, or as medians (25th–75th percentile) for variables with non-normal distribution before transformation. A *p*-value < 0.05 was considered statistically significant.

## 3. Results

### 3.1. Effects of Vitamin B6 and Folic Acid on the Body Weight and Cardio-Somatic Index of Experimental Animals

**Figure 2:**
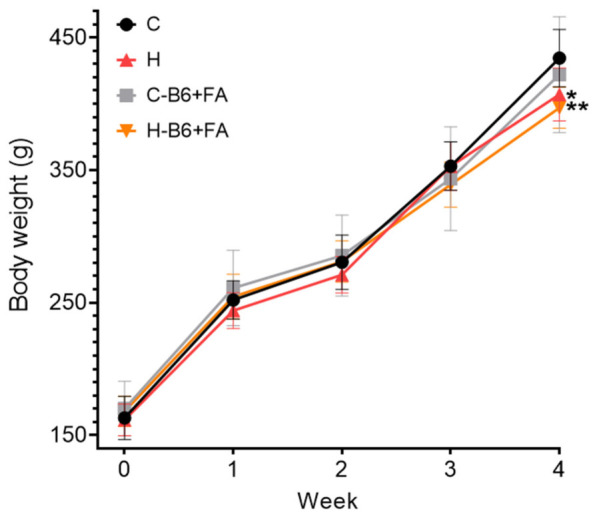
Effects of vitamin B6 and folic acid on the body weight of experimental animals throughout the weeks of the experimental protocol. C: saline solution 0.2 mL/2x/day s.c. for 14 days + saline solution 0.5 mL/day i.p. for 28 days; H: homocysteine 0.45 µmol/g b.w./2x/day s.c. for 14 days + saline solution 0.5 mL/day i.p. for 28 days; C-B6+FA: saline solution 0.2 mL/2x/day s.c. for 14 days + vitamin B6 7 mg/kg b.w. and folic acid 5 mg/kg b.w. i.p. for 28 days; H-B6+FA: homocysteine 0.45 µmol/g b.w./2x/day s.c. for 14 days + vitamin B6 7 mg/kg b.w. and folic acid 5 mg/kg b.w. i.p. for 28 days. n = 10 per group. * *p* < 0.05 compared to the C group; ** *p* < 0.01 compared to the C group—Šidák post hoc test.

A significant difference in animal body weights among the different groups was present in the final week of the experimental cycle. Groups treated with Hcy had a lower body weight compared to the animals in the control group (C) (H, *p* < 0.05; H-B6+FA, *p* < 0.01) in the fourth week, at the end of the experimental protocol. Throughout all weeks of the experimental protocol, the animals in the C-B6+FA group and the animals in the H-B6+FA group did not differ significantly from each other in terms of body weight (Figure 2).

Animals treated with combined administration of vitamin B6 and FA did not show a significant difference in the cardio-somatic index (CSI) value compared to the animals in the C and H groups.

### 3.2. Effects of Vitamin B6 and Folic Acid on Cardiovascular and Hemostatic Biomarkers in Serum and Plasma

**Figure 3:**
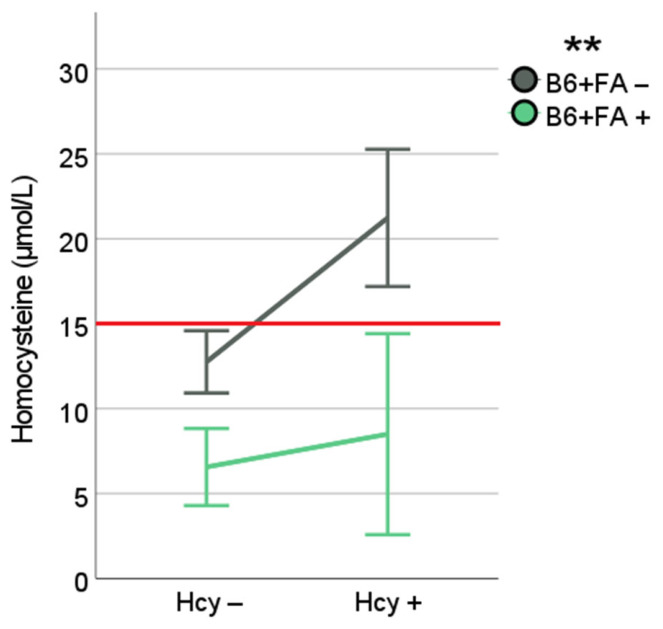
Effects of vitamin B6 and folic acid on homocysteine concentration in serum. B6+FA+: rats treated with vitamin B6 7 mg/kg b.w. and folic acid 5 mg/kg b.w. i.p. for 28 days; B6+FA−: rats treated with saline solution 0.5 mL/day i.p. for 28 days; Hcy+: rats treated with homocysteine 0.45 µmol/g b.w./2x/day s.c. for 14 days; Hcy−: rats treated with saline solution 0.2 mL/2x/day s.c. for 14 days. The red line represents the threshold value for hyperhomocysteinemia (15 µmol/L). Combined B6+FA supplementation was the dominant factor associated with lower serum Hcy concentrations, irrespective of Hcy loading status. ** *p* < 0.01—two-way ANOVA.

Factorial analysis of serum and plasma biomarkers revealed that the most prominent effects were related to serum Hcy, LDL, triglycerides, and vitamin B12. The serum Hcy concentration was markedly influenced by B6+FA supplementation, with a significant main effect of B6+FA (*p* < 0.001) (Figure 3), whereas the main effect of Hcy was not significant (*p* = 0.325), and no significant Hcy × B6+FA interaction was detected (*p* = 0.120). At the group level, serum Hcy was highest in the H group (21.2 ± 4.0 µmol/L), whereas both supplemented groups showed substantially lower values—namely, 6.6 ± 2.3 µmol/L in the C-B6+FA group and 8.5 ± 5.9 µmol/L in the H-B6+FA group. This pattern indicates that combined B6+FA supplementation was the dominant factor associated with lower serum Hcy concentrations, irrespective of Hcy loading status (Figure 3). The graphical presentation similarly showed a clear downward shift in Hcy values in both vitamin-treated groups, including the H-B6+FA group, in which serum Hcy remained below the hyperhomocysteinemic range despite exogenous Hcy administration.

A similar pattern was observed for the serum LDL concentration. The two-way ANOVA showed a significant main effect of B6+FA supplementation (*p* < 0.001), while the main effect of Hcy (*p* = 0.272) and the Hcy × B6+FA interaction (*p* = 0.120) were not significant. LDL values were highest in the H group (0.26 ± 0.10 mmol/L), whereas lower values were observed in both vitamin-treated groups, particularly in the H-B6+FA group (0.09 ± 0.07 mmol/L), as well as the C-B6+FA group (0.13 ± 0.09 mmol/L) (Table 1). Thus, the decrease in LDL appears to be primarily attributable to B6+FA supplementation rather than to Hcy loading itself or to a specific interaction between the two factors. On the graph/table pattern, this is reflected as a generalized reduction in LDL values in the supplemented groups compared with the non-supplemented, Hcy-loaded condition.

The triglyceride concentration showed a significant Hcy × B6+FA interaction (*p* = 0.047), whereas neither the main effect of Hcy (*p* = 0.315) nor the main effect of B6+FA (*p* = 0.378) was significant. This indicates that the effect of one factor depended on the level of the other factor. Specifically, in the absence of vitamin supplementation, Hcy loading was associated with a decrease in TG values from 0.77 ± 0.17 mmol/L in the C group to 0.57 ± 0.10 mmol/L in the H group. However, in the presence of B6+FA supplementation, this pattern was not maintained, as TG values were 0.69 ± 0.29 mmol/L in the C-B6+FA group and 0.76 ± 0.20 mmol/L in the H-B6+FA group. Therefore, the interaction reflects opposite or divergent directional changes in TG depending on whether B6+FA supplementation was present. This result should be described as an interaction effect rather than as an isolated effect of Hcy or B6+FA.

Vitamin B12 showed a more complex pattern, with significant main effects of both Hcy (*p* = 0.002) and B6+FA supplementation (*p* = 0.002), as well as a highly significant Hcy × B6+FA interaction (*p* < 0.001). The vitamin B12 concentration was markedly increased in the H group (627.2 ± 81.3 pg/mL) compared with the control group (460.2 ± 50.0 pg/mL). However, this increase was not observed in the supplemented groups, where vitamin B12 values were 486.4 ± 68.2 pg/mL in C-B6+FA and 463.5 ± 66.1 pg/mL in H-B6+FA. This pattern indicates that Hcy loading increased serum vitamin B12 only in the absence of B6+FA supplementation, whereas combined supplementation prevented or abolished this increase. Therefore, the significant interaction suggests that the effect of Hcy on the vitamin B12 concentration was dependent on B6+FA status.

### 3.3. Effects of Vitamin B6 and Folic Acid on Oxidative Stress Parameters in Heart Tissue

**Figure 4:**
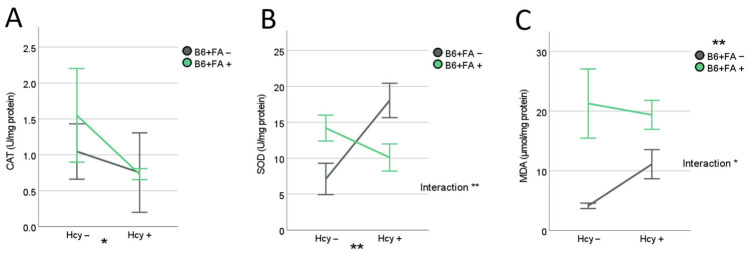
Effects of vitamin B6 and folic acid on oxidative stress parameters in cardiac tissue: catalase activity (**A**), superoxide dismutase activity (**B**), and malondialdehyde concentration (**C**). B6+FA+: rats treated with vitamin B6 7 mg/kg b.w. and folic acid 5 mg/kg b.w. i.p. for 28 days; B6+FA−: rats treated with saline solution 0.5 mL/day i.p. for 28 days; Hcy+: rats treated with homocysteine 0.45 µmol/g b.w./2x/day s.c. for 14 days; Hcy−: rats treated with saline solution 0.2 mL/2x/day s.c. for 14 days. * *p* < 0.05; ** *p* < 0.01—two-way ANOVA.

In cardiac tissue homogenates, CAT activity was significantly affected by Hcy loading, with a significant main effect of Hcy (*p* = 0.018), whereas the main effect of B6+FA (*p* = 0.269) and the Hcy × B6+FA interaction (*p* = 0.229) were not significant (Table 2). CAT activity was 1.046 ± 0.385 U/mg protein in the C group and 0.753 ± 0.553 U/mg protein in the H group, while values in the supplemented groups were 1.550 ± 0.652 U/mg protein in C-B6+FA and 0.731 ± 0.077 U/mg protein in H-B6+FA. The graphical trend indicates lower CAT activity in Hcy-loaded groups compared with their corresponding non-Hcy groups, particularly when comparing the C-B6+FA and H-B6+FA groups. Since the interaction was not significant, this pattern should be interpreted primarily as an Hcy-related effect rather than as evidence that B6+FA significantly modified the effect of Hcy on CAT activity (Figure 4A).

SOD activity showed a significant main effect of Hcy (*p* = 0.002) and a highly significant Hcy × B6+FA interaction (*p* < 0.001), while the main effect of B6+FA was not significant (*p* = 0.651) (Table 2). SOD activity increased markedly in the H group (18.052 ± 2.398 U/mg protein) compared with the C group (7.116 ± 2.187 U/mg protein), indicating a strong Hcy-associated increase in antioxidant enzyme activity. However, in the H-B6+FA group, SOD activity was lower (10.103 ± 1.891 U/mg protein) than in the H group, while the C-B6+FA group showed an intermediate value (14.207 ± 1.804 U/mg protein). Thus, the significant interaction indicates that the effect of Hcy on SOD activity depended on the presence of B6+FA supplementation. Graphically, the pattern is characterized by a steep increase in SOD activity in response to Hcy in the absence of supplementation, whereas this Hcy-associated increase is attenuated when B6+FA is co-administered (Figure 4B).

MDA concentration in cardiac tissue was significantly affected by B6+FA supplementation (*p* < 0.001) and showed a significant Hcy × B6+FA interaction (*p* = 0.010), while the main effect of Hcy alone was not significant (*p* = 0.112) (Table 2). MDA values were lowest in the C group (4.143 ± 0.454 µmol/mg protein); increased in the H group (11.116 ± 2.437 µmol/mg protein); and highest in the vitamin-supplemented groups, particularly C-B6+FA (21.280 ± 5.799 µmol/mg protein) and H-B6+FA (19.382 ± 2.424 µmol/mg protein). The graphical presentation therefore shows a pronounced upward shift in MDA values in both B6+FA-treated groups. However, because the interaction was also significant, the effect of B6+FA on lipid peroxidation was not completely uniform across Hcy conditions. Overall, these findings indicate that B6+FA supplementation was associated with an increased MDA concentration in cardiac tissue, with the magnitude of this effect depending partly on Hcy status (Figure 4C).

### 3.4. Effects of Vitamin B6 and Folic Acid on Metabolic Enzyme Activities in Heart Tissue

**Figure 5:**
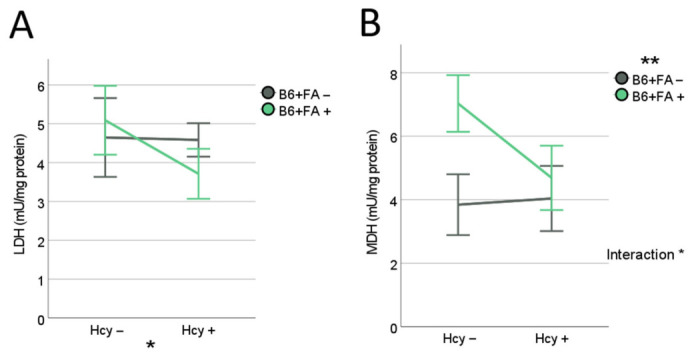
Effects of vitamin B6 and folic acid on metabolic enzyme activities in heart tissue: lactate dehydrogenase activity (**A**), and malate dehydrogenase activity (**B**). B6+FA+: rats treated with vitamin B6 7 mg/kg b.w. and folic acid 5 mg/kg b.w. i.p. for 28 days; B6+FA−: rats treated with saline solution 0.5 mL/day i.p. for 28 days; Hcy+: rats treated with homocysteine 0.45 µmol/g b.w./2x/day s.c. for 14 days; Hcy−: rats treated with saline solution 0.2 mL/2x/day s.c. for 14 days. * *p* < 0.05; ** *p* < 0.01—two-way ANOVA.

LDH activity in heart tissue was not significantly affected by B6+FA supplementation (*p* = 0.546), and neither the main effect of Hcy (*p* = 0.055) nor the Hcy × B6+FA interaction (*p* = 0.076) reached statistical significance (Figure 5A).

MDH activity showed significant main effects of both Hcy (*p* = 0.025) and B6+FA supplementation (*p* < 0.001), together with a significant Hcy × B6+FA interaction (*p* = 0.010). MDH activity was similar in the C and H groups (3.844 ± 0.956 and 4.039 ± 1.026 mU/mg protein, respectively) but increased markedly in the C-B6+FA group (7.032 ± 0.893 mU/mg protein). In contrast, this increase was much less pronounced in the H-B6+FA group (4.689 ± 1.013 mU/mg protein). Thus, B6+FA supplementation increased MDH activity primarily in animals not exposed to Hcy, whereas Hcy loading attenuated this B6+FA-associated increase. The significant interaction therefore indicates that the metabolic effect of B6+FA on MDH activity was dependent on Hcy status (Figure 5B).

### 3.5. Effects of Vitamin B6 and Folic Acid on Histological Changes and Histomorphometric Parameters of the Heart and Aortic Wall Tissue

**Figure 6:**
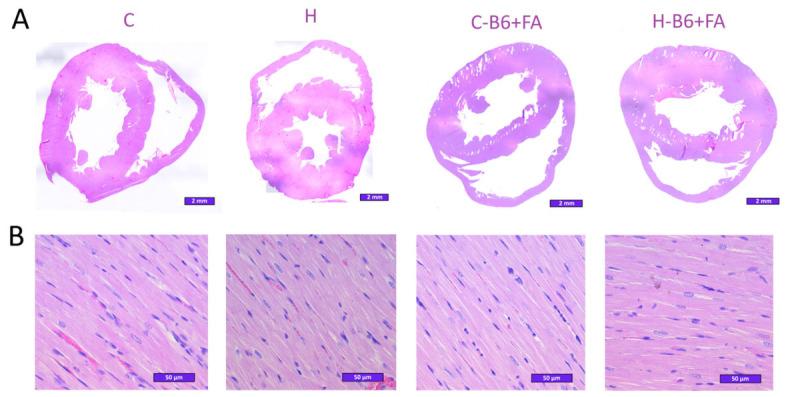
Transverse section of the heart—hematoxylin/eosin staining ((**A**) magnification ×50; (**B**) magnification ×400). C: saline solution 0.2 mL/2x/day s.c. for 14 days + saline solution 0.5 mL/day i.p. for 28 days; H: homocysteine 0.45 µmol/g b.w./2x/day s.c. for 14 days + saline solution 0.5 mL/day i.p. for 28 days; C-B6+FA: saline solution 0.2 mL/2x/day s.c. for 14 days + vitamin B6 7 mg/kg b.w. and folic acid 5 mg/kg b.w. i.p. for 28 days; H-B6+FA: homocysteine 0.45 µmol/g b.w./2x/day s.c. for 14 days + vitamin B6 7 mg/kg b.w. and folic acid 5 mg/kg b.w. i.p. for 28 days. n = 5 per group.

Histological examination of the heart showed preserved myocardial organization in all experimental groups. Cardiomyocytes retained physiological morphology, and no areas of necrosis or connective tissue proliferation were observed (Figure 6). However, histomorphometric analysis revealed treatment-related changes in ventricular wall thickness (Table 3).

Left ventricular wall thickness showed a significant main effect of B6+FA supplementation (*p* = 0.024), whereas the Hcy effect approached but did not reach statistical significance (*p* = 0.060), and the interaction was not significant (*p* = 0.767). Group values increased from 2110.8 ± 62.3 µm in the C group to 2307.1 ± 133.1 µm in the H group, 2362.8 ± 313.2 µm in the C-B6+FA group, and 2510.9 ± 205.9 µm in the H-B6+FA group. This pattern suggests an overall tendency toward greater left ventricular wall thickness in supplemented animals, particularly in the combined H-B6+FA group, although the absence of a significant interaction indicates that this effect should not be interpreted as a specific synergistic effect of Hcy and B6+FA.

Right ventricular wall thickness showed significant main effects of both Hcy (*p* = 0.019) and B6+FA supplementation (*p* = 0.002), as well as a significant Hcy × B6+FA interaction (*p* = 0.005). Right ventricular wall thickness increased from 790.2 ± 45.3 µm in the C group to 1025.4 ± 146.9 µm in the H group, indicating Hcy-associated right ventricular thickening. However, this increase was not present in the supplemented groups, where values were 777.1 ± 59.4 µm in C-B6+FA and 756.6 ± 51.7 µm in H-B6+FA. The graphical pattern therefore shows a marked increase in the H group, followed by normalization or reduction in right ventricular wall thickness in the H-B6+FA group. Because the interaction was significant, these findings indicate that B6+FA supplementation modified the effect of Hcy on right ventricular wall thickness and appeared to prevent the Hcy-associated increase in this parameter.

Interventricular septum thickness was significantly affected by Hcy loading (*p* < 0.001), while the main effect of B6+FA (*p* = 0.888) and the interaction (*p* = 0.485) were not significant. Septal thickness increased from 1650.4 ± 132.1 µm in the C group to 1972.2 ± 263.0 µm in the H group and from 1604.0 ± 123.8 µm in the C-B6+FA group to 2042.0 ± 173.5 µm in the H-B6+FA group. This indicates a consistent Hcy-associated increase in septal thickness across both supplementation conditions. Since the interaction was not significant, B6+FA did not significantly modify the effect of Hcy on interventricular septum thickness.

The transverse diameter of cardiomyocytes did not show significant main effects of Hcy (*p* = 0.081) or B6+FA (*p* = 0.177), nor a significant interaction (*p* = 0.184).

**Figure 7:**
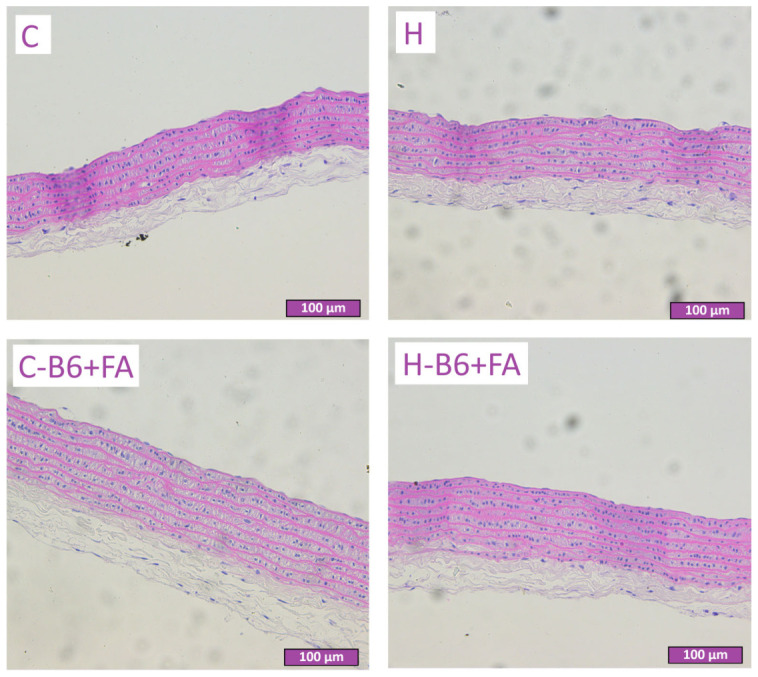
Transverse section of the aorta—hematoxylin/eosin staining (magnification x200). C: saline solution 0.2 mL/2x/day s.c. for 14 days + saline solution 0.5 mL/day i.p. for 28 days; H: homocysteine 0.45 µmol/g b.w./2x/day s.c. for 14 days + saline solution 0.5 mL/day i.p. for 28 days; C-B6+FA: saline solution 0.2 mL/2x/day s.c. for 14 days + vitamin B6 7 mg/kg b.w. and folic acid 5 mg/kg b.w. i.p. for 28 days; H-B6+FA: homocysteine 0.45 µmol/g b.w./2x/day s.c. for 14 days + vitamin B6 7 mg/kg b.w. and folic acid 5 mg/kg b.w. i.p. for 28 days. n = 5 per group.

Histomorphometric parameters of the aortic wall were not significantly affected by Hcy loading, B6+FA supplementation, or their interaction (Figure 7).

## 4. Discussion

This study investigated the effects of vitamin B6 and FA on cardiometabolic biomarkers, cardiac oxidative stress, enzyme activities, and cardiovascular histomorphometric parameters in Hcy-loaded rats.

Biochemical analysis of cardiometabolic biomarkers in serum and plasma demonstrated that the serum Hcy concentration was primarily influenced by B6+FA supplementation, with a significant main effect of B6+FA, while the main effect of Hcy and the Hcy × B6+FA interaction were not significant. This suggests that combined supplementation reduced serum Hcy concentrations independently of Hcy-loading status. These findings align with the previously described roles of vitamin B6 and FA as cofactors of enzymes involved in the remethylation and trans-sulfuration metabolic pathways [21]. Similar findings were observed in our previous study that investigated the protective effect of vitamin B6 administered alone under conditions of homocysteine loading in rats, where it was noted that animals treated with vitamin B6 in combination with homocysteine did not develop hyperhomocysteinemia [35]. Results of our study indicate that the LDL concentration showed a significant main effect of B6+FA, whereas the Hcy effect and the Hcy × B6+FA interaction were not significant, indicating that the LDL-lowering pattern was mainly attributable to vitamin supplementation rather than to a specific interaction with Hcy loading. Some studies have shown that elevated Hcy levels are associated with higher LDL levels in patients with hypothyroidism, with authors noting that elevated plasma levels of Hcy promoted LDL, suggesting that HHcy may contribute to dyslipidemia [36]. Furthermore, a positive correlation has been reported between Hcy levels and the presence of hyperlipidemia. In the overall population, HHcy was associated with an increased risk of hypercholesterolemia. Among females, HHcy was linked to an elevated risk of high-LDL hyperlipidemia, while in middle-aged individuals, HHcy was associated with an increased risk of both high-triglyceride and high-LDL hyperlipidemia [37]. In our study, triglyceride concentrations showed a significant Hcy × B6+FA interaction, despite non-significant main effects of Hcy and B6+FA, suggesting that the direction of TG change depended on whether Hcy loading and vitamin supplementation were combined. In a study examining the effects of combined vitamin B6 and FA administration in a monocrotaline-induced model of right ventricular failure, the combined treatment did not produce significant alterations in lipid status relative to the control group [38]. Although the literature suggests that there may be an association between elevated homocysteine levels and cardiac troponins across different clinical contexts, implying that their combined assessment could potentially contribute to cardiovascular risk stratification [39], our results showed no significant treatment-related effects on hs-TnT, total cholesterol, HDL, serum LDH, folate, fibrinogen, or vWF, whereas vitamin B12 displayed significant main effects of both Hcy and B6+FA and a significant Hcy × B6+FA interaction, indicating a factor-dependent response. The lower vitamin B12 levels in the B6+FA-treated groups may be related to increased utilization of vitamin B12 as a cofactor for methionine synthase during Hcy remethylation.

Our study did not measure concentrations of pro-inflammatory cytokines such as IL-1β, TNF-α, and IL-6. However, data from the literature suggest that supplementation with vitamin B6 and folic acid can significantly influence their levels. Mouse studies have shown that B6 can reduce serum IL-1β, TNF-α, and IL-6 following LPS-induced endotoxemia by inhibiting the activation of NF-κB and mitogen-activated protein kinase signaling pathways, which are crucial for cytokine production during inflammation, indicating anti-inflammatory effects in vivo [40]. Vitamin B6—particularly its active form, pyridoxal 5’-phosphate—has been shown to prevent IL-1β production by modulating the nucleotide-binding oligomerization domain and leucine-rich repeat and pyrin domain-containing protein 3 inflammasome activation and inhibiting its signaling, further explaining how B6 can reduce this pro-inflammatory cytokine [41]. Conversely, vitamin B6 deficiency can lead to increased IL-1β production and tissue damage due to elevated ROS [42], highlighting that adequate B6 levels exert anti-inflammatory effects. On the other hand, a study in rats treated with lipopolysaccharide to induce neuroinflammation showed that folic acid supplementation (5–20 mg/kg/day) significantly reduced hippocampal levels of IL-6 and IL-1β, which were increased by lipopolysaccharide, and attenuated oxidative stress markers such as malondialdehyde while improving antioxidant defenses like SOD activity [43]. This indicates that folic acid can lower pro-inflammatory cytokines in vivo in animal inflammation models. In addition, cell culture models directly examining inflammatory signaling under stress conditions relevant to hypoxic or oxidative environments have shown that folic acid pretreatment of THP-1 cells decreased IL-1β and TNF-α protein and mRNA levels induced by hypoxia while simultaneously increasing anti-inflammatory cytokine IL-10 [44]. These effects were mediated through the inhibition of the PI3K/Akt/HIF-1α pathway, a key regulator of inflammatory gene expression under stress [44]. Furthermore, in a randomized dietary intervention study, increased folate intake from foods over eight weeks significantly reduced TNF-α, IL-6, and IL-1β levels in overweight or obese women carrying the methylenetetrahydrofolate reductase C677T genotype, particularly in those with the TT variant [45]. While this was a dietary folate study rather than direct supplementation, it suggests that folate status can modulate inflammatory cytokines in humans, with effects potentially influenced by the genetic background. Collectively, these animal, cellular, and human data support the notion that folic acid can exert anti-inflammatory effects by lowering pro-inflammatory cytokines and improving antioxidant defenses under conditions of stress or elevated inflammatory signaling.

Results of our study indicate that CAT activity was characterized by a significant main effect of Hcy, without a significant B6+FA effect or interaction. In contrast, SOD activity showed a significant main effect of Hcy, together with a highly significant Hcy × B6+FA interaction, indicating that the Hcy-associated increase in SOD activity was modified by vitamin supplementation. MDA concentration showed a significant main effect of B6+FA and a significant Hcy × B6+FA interaction, suggesting that the increase in lipid peroxidation was mainly associated with supplementation but varied according to Hcy status.

Elevated Hcy levels have been consistently shown to enhance oxidative stress by promoting mitochondrial dysfunction and ROS production in various experimental models. In a rat model of myocardial ischemia/reperfusion injury, Hcy treatment significantly increased ROS generation, disrupted mitochondrial function, and exacerbated oxidative damage [46]. Similarly, Hcy exposure reduced mitochondrial membrane potential and elevated ROS levels in human trabecular meshwork cells, demonstrating dose-dependent oxidative stress in vitro [47]. In aging rats with induced HHcy, cardiac and systemic oxidative markers were elevated while antioxidant enzyme activities declined, highlighting redox imbalance [8]. Hcy also increased intracellular ROS and promoted inflammasome-linked oxidative responses in macrophages, indicating a role in immune-cell oxidative dysregulation [48].

In contrast, evidence indicates that supplementation with FA and vitamin B6 can ameliorate oxidative stress associated with HHcy. In rats treated with Hcy, vitamin B6 supplementation normalized oxidative stress biomarkers and restored vascular prostacyclin production, suggesting antioxidative effects [49]. Meta-analytic data reveal that FA supplementation enhances total antioxidant capacity and glutathione levels, which are inversely linked to oxidative stress [50]. Clinical trials demonstrate that combined FA and B-vitamin treatment significantly reduces plasma Hcy, a known promoter of oxidative stress [51]. However, some clinical work has found no significant associations between folate or B6 status and oxidative stress markers, suggesting context-dependent effects [52]. On the other hand, in our study, supplementation with vitamin B6 and folic acid led to a significant increase in the lipid peroxidation index, and this finding is described more specifically as a significant B6+FA-related increase in cardiac MDA concentration accompanied by a significant Hcy × B6+FA interaction, indicating that the magnitude of lipid peroxidation depended partly on Hcy status. This change was not observed in our previous study, in which vitamin B6 supplementation under Hcy-loading conditions resulted in a decrease in the lipid peroxidation index [35]. To the best of our knowledge, there are no published human or mammalian studies showing that folic acid or vitamin B6 supplementation directly increases lipid peroxidation markers such as MDA. However, experimental evidence from non-mammalian models suggests that very high doses of folic acid may disrupt one-carbon metabolism and induce oxidative stress, indicating that extremely high folate exposure can, itself, be pro-oxidant under some conditions [53]. High concentrations of FA in vitro were shown to induce oxidative stress, acute cytotoxicity, and long-term fibrogenic changes in kidney epithelial cells (human and mouse lines) [54]. Specifically, high-dose FA significantly increased intracellular ROS, altered the expression of antioxidant- (glutathione peroxidase and manganese-SOD) and DNA damage-related genes, and upregulated markers of epithelial-to-mesenchymal transition and fibrosis. These effects occurred at supraphysiological FA concentrations, highlighting that excessive folate exposure can, itself, act as a pro-oxidant. This mechanistic evidence provides a plausible explanation for transient increases in lipid peroxidation observed under certain experimental conditions. Moreover, the high heterogeneity of responses across studies indicates that the effects of supplementation on oxidative markers can vary with dose, metabolic context, and redox balance, including subgroup differences in health status, sex, and duration of intervention, even within human clinical trials [50].

MDH activity showed significant main effects of both Hcy and B6+FA, as well as a significant Hcy × B6+FA interaction. This pattern indicates that B6+FA increased MDH activity mainly in non-Hcy-loaded animals, whereas Hcy loading attenuated this supplementation-associated increase. Previous studies support the concept that Hcy does not necessarily alter MDH activity when applied independently; in our earlier work, independent Hcy application did not change total MDH activity, whereas Hcy combined with aerobic physical activity increased mitochondrial MDH activity, suggesting a compensatory mitochondrial response under conditions of increased metabolic demand [55]. Tatarková et al. [56] noted that cytosolic MDH participates, together with mitochondrial MDH, in the malate–aspartate shuttle and that the activities of both cytosolic and mitochondrial MDH isoforms were previously reported to remain unaltered in the heart of hyperhomocysteinemic rats. In this study, LDH activity did not show significant main or interaction effects.

Data from the literature indicate that Hcy loading can influence the activity of enzymes involved in energy metabolism. By analyzing the effects of HHcy in humans and animal models, a study by Cueto et al. [57] reported reduced expression of 15 nuclear genes encoding mitochondrial electron transport chain (METC) proteins under HHcy conditions. Among the identified genes, eleven encoded subunits of METC complex I, one encoded a subunit of complex IV, and two encoded subunits of complex V. Because four of the eleven complex I genes downregulated by Hcy encode core subunits essential for enzymatic activity, the authors suggested that complex I dysfunction resulting from altered gene expression may play a primary role in Hcy-induced disturbances of energy metabolism. This study also demonstrated that the heart and brain are particularly sensitive to the effects of HHcy on energy metabolism.

An imbalance among METC complexes may lead to increased production of reactive oxygen species (ROS) and oxidative damage. Substantial evidence of HHcy-associated mitochondrial oxidative injury has been reported across various tissues under diverse experimental conditions. Unlike nuclear deoxyribonucleic acid (DNA), mitochondrial DNA lacks histone protection and possesses less efficient repair mechanisms, rendering it more susceptible to oxidative damage and mutations [58]. Proteins represent primary targets of ROS, and HHcy-induced protein modifications—such as sulfhydryl group oxidation, tyrosine nitration, and carbonyl group formation—have been documented in cardiac, cerebral, and vascular tissues [58,59,60]. These protein modifications lead to structural alterations and functional loss, ultimately disrupting the homeostasis of energy metabolism.

HHcy impacts mitochondrial homeostasis by disrupting biogenesis and quality control mechanisms. Hcy exposure was associated with downregulation of mitochondrial biogenesis regulators, including peroxisome proliferator-activated receptor gamma coactivator 1-alpha, nuclear respiratory factor 1, and mitochondrial transcription factor A—transcriptional factors critical for maintaining mitochondrial DNA (mtDNA) replication, the expression of METC components, and overall mitochondrial mass [61]. These changes collectively impair mitochondrial biogenesis and contribute to deficits in energy metabolism and cellular resilience under stress. Moreover, inhibition of the NAD^+^/Sirtuin 1 signaling axis was implicated in these effects, as Hcy decreased Sirt1 activity by interfering with NAD^+^ metabolism, further suppressing peroxisome proliferator-activated receptor gamma coactivator 1-alpha-mediated transcriptional programs and mitophagy [61]. In endothelial cells, Hcy treatment led to morphological changes in mitochondria, loss of mitochondrial membrane potential, decreased ATP content, and impaired expression of mitochondrial dynamics proteins such as dynamin-related protein 1 and mitofusin-2, indicating an imbalance between mitochondrial fission and fusion. Hcy also increased mitochondrial calcium uptake through enhanced mitochondria-associated membranes and activation of the mitochondrial calcium uniporter, suggesting that calcium overload contributes to mitochondrial dysfunction and ROS generation [62].

In this study, left ventricular wall thickness showed a significant main effect of B6+FA, without a significant interaction, while right ventricular wall thickness showed significant main effects of Hcy and B6+FA, together with a significant Hcy × B6+FA interaction. This supports the interpretation that B6+FA supplementation modified the Hcy-associated increase in right ventricular wall thickness. Interventricular septum thickness showed a significant main effect of Hcy, whereas cardiomyocyte transverse diameter and all examined aortic histomorphometric parameters remained without significant main or interaction effects.

Elevated Hcy has been shown to induce structural and functional changes in cardiac and vascular tissues. Experimental studies demonstrate that Hcy promotes cardiomyocyte apoptosis, increases ROS, and impairs mitochondrial function, contributing to cardiac dysfunction and remodeling [63]. Chronic HHcy can induce adverse cardiac remodeling characterized by fibrosis, increased collagen deposition, and reduced contractility, mediated in part by matrix metalloproteinase (MMP)/tissue inhibitor of metalloproteinases (TIMP) imbalance [64]. In vascular tissues, Hcy can stimulate vascular smooth-muscle-cell proliferation and migration, promotes inflammatory activation, and drives aortic remodeling [65]. Hcy can also activate profibrotic pathways, including by transforming growth factor-β and Akt signaling, further exacerbating myocardial fibrosis [66].

Supplementation with FA and B vitamins has been shown to mitigate Hcy-induced remodeling. FA can prevent cardiac dysfunction and reduces myocardial fibrosis, likely by normalizing TIMP levels and enhancing Hcy metabolism [67,68]. These findings suggest that B-vitamin supplementation may counteract Hcy-induced structural and functional alterations in cardiac and vascular tissues. Trimarchi et al. emphasized that inflammatory biomarkers such as the C-reactive protein-to-albumin ratio, lymphocyte-to-C-reactive protein ratio, C-reactive protein-to-hemoglobin ratio, and neutrophil-to-C-reactive protein ratio correlate with heart-failure severity. Although inflammatory markers were not directly assessed in the present study, the observed Hcy-related changes in cardiac oxidative stress parameters and right ventricular wall thickness suggest that future studies should examine whether B6+FA supplementation modulates the oxidative–inflammatory axis involved in Hcy-associated cardiac remodeling.

Evidence from experimental models indicates that elevated Hcy impairs myocardial contractile performance and recovery after ischemic stress in perfused hearts. Acute exposure to clinically relevant Hcy levels (0.1 mM) in isolated rat hearts reduced the rate-pressure product during reperfusion after global ischemia, indicating impaired contractile function and poorer recovery after ischemia [69]. In vivo, FA supplementation has been shown to improve left ventricular systolic function, ejection fraction, and fractional shortening in high-fat diet-induced cardiac dysfunction models [67]. FA supplementation prevented age-associated left ventricular hypertrophy and preserved cardiac function while reducing fibrosis, apoptosis, and oxidative stress in the myocardium [70]. Additionally, reviews of experimental myocardial infarction and heart-failure models support the idea that B-vitamin supplementation can enhance vasodilatation and coronary flow and reduce oxidative stress, further implying improved functional performance [71]. These findings collectively suggest that functional parameters of the heart can be positively modulated by folic acid and, potentially, by vitamin B6 and FA in cardiac stress.

Although our study offers valuable insights into the effects of Hcy, vitamin B6, and FA on oxidative stress markers, metabolic enzyme activities, and histomorphological features in cardiac tissue, several limitations should be noted. Key inflammatory markers (e.g., tumor necrosis factor-α, IL-1, and IL-6), signaling pathways (NF-κB and mitogen-activated protein kinase), and mitochondrial gene expression were not assessed, limiting the interpretation of pathophysiological mechanisms. Therefore, any interpretation regarding anti-inflammatory mechanisms remains speculative. Immunohistochemical analyses and physiological measurements of cardiac function (e.g., via the Langendorff system) were also lacking, restricting spatial and functional evaluation of oxidative damage and contractility. Future studies integrating transcriptomic, proteomic, and immunohistopathological approaches are warranted to better elucidate the interplay between Hcy, oxidative stress, and cardiac pathophysiology.

A limitation of the present study is that the effects of Hcy loading and combined vitamin B6 and folic acid supplementation were examined only in juvenile male Wistar albino rats. This experimental approach was chosen to obtain a relatively homogeneous cohort and to assess early cardiovascular and metabolic responses during a period of active postnatal growth; however, both sex and developmental stage should be considered important biological variables when interpreting the results. Previous studies have demonstrated sex-dependent responses to HHcy. In spontaneously hypertensive rats, methionine-induced HHcy produced comparable increases in serum Hcy in the two sexes, but female rats showed attenuated blood pressure development and enhanced endothelium-dependent relaxation compared with males [72]. More recently, sex-specific cardiac responses to chronic mild HHcy were reported in Wistar rats, with males showing pronounced increases in ROS, TBARS, CAT activity, NFκB signaling, and pro-inflammatory cytokines, whereas females showed reductions in GPx activity and nitrite levels, together with increased IL-6 and RELA expression [73]. In addition, in a rat model of L-methionine-induced HHcy, Milyutina et al. reported that aging, rather than HHcy alone, was a major determinant of biogenic amine content in hypothalamic structures, while oxidative stress-related changes appeared to be partly compensated for by antioxidant defense mechanisms [74]. Therefore, the present findings should be interpreted within the biological context of juvenile male animals, and future studies should include both sexes and different age groups to determine whether the cardiovascular effects of Hcy loading and B6+FA supplementation are sex- and age-dependent.

A limitation of the present study is that the sample size was not determined using a formal power analysis tool but was based on previous studies with similar experimental designs, feasibility considerations, and the ethical principle of reducing animal use. In addition, biochemical and histological measurements were not performed under blinded conditions. These aspects should be considered methodological limitations, and future studies should include prospective sample-size calculation and blinded outcome assessment.

## 5. Conclusions

The findings of this study indicate that combined vitamin B6 and folic acid supplementation significantly modulates several biochemical and structural consequences of Hcy loading in rats. B6+FA markedly reduced serum Hcy and LDL concentrations, suggesting a favorable effect on Hcy metabolism and the lipid profile. In cardiac tissue, Hcy loading was associated with increased SOD activity and right ventricular wall thickness, while combined B6+FA treatment attenuated these alterations, indicating a potential protective effect against Hcy-related oxidative and structural cardiac changes. However, supplementation was also associated with increased cardiac MDA levels, suggesting enhanced lipid peroxidation and emphasizing that its effects on redox balance may be complex. MDH activity was increased mainly in non-Hcy-loaded supplemented animals, whereas LDH activity and aortic histomorphometric parameters remained unchanged. Overall, B6+FA supplementation appears to exert beneficial cardiometabolic and cardiac remodeling effects, although its potential pro-oxidative impact warrants cautious interpretation and further investigation.

## Figures and Tables

**Table 1:** Effects of vitamin B6 and folic acid on cardiovascular and hemostatic biomarkers in serum and plasma.

Parameter	Groups				Source of Variation (*p* Value)		
	C	H	C-B6+FA	H-B6+FA	Hcy	B6+FA	Interaction
Hcy (μmol/L) s	12.7 ± 1.8	21.2 ± 4.0	6.6 ± 2.3	8.5 ± 5.9	0.325	<0.001 **	0.120
CHOL (mmol/L) s	1.76 (1.54–1.93)	1.81 (1.58–2.00)	1.56 (1.24–1.68)	1.58 (1.49–2.07)	0.204	0.054	0.439
HDL (mmol/L) s	1.25 (1.12–1.40)	1.29 (1.15–1.42)	1.20 (1.02–1.36)	1.27 (1.24–1.64)	0.113	0.961	0.325
LDL (mmol/L) s	0.16 ± 0.09	0.26 ± 0.10	0.13 ± 0.09	0.09 ± 0.07	0.272	<0.001 **	0.120
TG (mmol/L) s	0.77 ± 0.17	0.57 ± 0.10	0.69 ± 0.29	0.76 ± 0.20	0.315	0.378	0.047 *
LDH (U/L) s	3546 ± 1305	3323 ± 666	3257 ± 666	2865 ± 604	0.362	0.235	0.517
hs-TnT (ng/L) s	22.0 (16.0–53.0)	21.5 (13.7–39.2)	24.0 (17.2–38.7)	15.5 (14.0–61.2)	0.787	0.974	0.601
Vitamin B12 (pg/mL) s	460.2 ± 50.0	627.2 ± 81.3	486.4 ± 68.2	463.5 ± 66.1	0.002 **	0.002 **	<0.001 **
Folate (ng/mL) s	19.2 (19.0–20.0)	20.0 (20.0–20.0)	20.0 (20.0–20.0)	20.0 (20.0–20.0)	0.535	0.676	0.066
Fibrinogen (g/L) p	1.9 ± 0.4	1.8 ± 0.1	1.9 ± 0.2	1.7 ± 0.2	0.141	0.469	0.641
D-dimer < 0.17 mg/L p	10 (100%)	10 (100%)	10 (100%)	10 (100%)	/	/	/
vWF (%) p	155.0 (122.2–167.5)	151.0 (144.5–166.5)	151.0 (145.2–151.0)	151.0 (144.7–151.0)	0.349	0.902	0.242

Hcy—homocysteine; CHOL—total cholesterol; HDL—high-density lipoprotein; LDL—low-density lipoprotein; TG—triglycerides; LDH—lactate dehydrogenase; hs-TnT—high-sensitivity troponin T; vWF—von Willebrand factor; s—value measured in serum; p—value measured in plasma. C: saline solution 0.2 mL/2x/day s.c. for 14 days + saline solution 0.5 mL/day i.p. for 28 days; H: homocysteine 0.45 µmol/g b.w./2x/day s.c. for 14 days + saline solution 0.5 mL/day i.p. for 28 days; C-B6+FA: saline solution 0.2 mL/2x/day s.c. for 14 days + vitamin B6 7 mg/kg b.w. and folic acid 5 mg/kg b.w. i.p. for 28 days; H-B6+FA: homocysteine 0.45 µmol/g b.w./2x/day s.c. for 14 days + vitamin B6 7 mg/kg b.w. and folic acid 5 mg/kg b.w. i.p. for 28 days. n = 10 per group. Data are presented as mean ± SD or median (25th–75th percentile) according to the data distribution.* *p* < 0.05; ** *p* < 0.01—two-way ANOVA.

**Table 2:** Effects of Hcy loading and B6+FA supplementation on oxidative stress markers and metabolic enzyme activities in heart-tissue homogenates.

Parameter	Groups				Source of Variation (*p* Values)		
	C	H	C-B6+FA	H-B6+FA	Hcy	B6+FA	Interaction
CAT (U/mg protein)	1.046 ± 0.385	0.753 ± 0.553	1.550 ± 0.652	0.731 ± 0.077	0.018 *	0.269	0.229
SOD (U/mg protein)	7.116 ± 2.187	18.052 ± 2.398	14.207 ± 1.804	10.103 ± 1.891	0.002 **	0.651	<0.001 **
MDA (umol/mg protein)	4.143 ± 0.454	11.116 ± 2.437	21.280 ± 5.799	19.382 ± 2.424	0.112	<0.001 **	0.010 *
LDH (mU/mg protein)	4.647 ± 1.015	4.585 ± 0.430	5.092 ± 0.887	3.712 ± 0.644	0.055	0.546	0.076
MDH (mU/mg protein)	3.844 ± 0.956	4.039 ± 1.026	7.032 ± 0.893	4.689 ± 1.013	0.025	<0.001 **	0.010 *

C: saline solution 0.2 mL/2x/day s.c. for 14 days + saline solution 0.5 mL/day i.p. for 28 days; H: homocysteine 0.45 µmol/g b.w./2x/day s.c. for 14 days + saline solution 0.5 mL/day i.p. for 28 days; C-B6+FA: saline solution 0.2 mL/2x/day s.c. for 14 days + vitamin B6 7 mg/kg b.w. and folic acid 5 mg/kg b.w. i.p. for 28 days; H-B6+FA: homocysteine 0.45 µmol/g b.w./2x/day s.c. for 14 days + vitamin B6 7 mg/kg b.w. and folic acid 5 mg/kg b.w. i.p. for 28 days. n = 10 per group. Data are presented as mean ± SD. * *p* < 0.05; ** *p* < 0.01—two-way ANOVA.

**Table 3:** Effects of Hcy loading and B6+FA supplementation on cardiac and aortic histomorphometric parameters.

Parameter	Groups				Source of Variation (*p* Values)		
	C	H	C-B6+FA	H-B6+FA	Hcy	B6+FA	Interaction
Left ventricular wall thickness (µm)	2110.8 ± 62.3	2307.1 ± 133.1	2362.8 ± 313.2	2510.9 ± 205.9	0.060	0.024 *	0.767
Right ventricular wall thickness (µm)	790.2 ± 45.3	1025.4 ± 146.9	777.1 ± 59.4	756.6 ± 51.7	0.019 *	0.002 **	0.005 **
Interventricular septum thickness (µm)	1650.4 ± 132.1	1972.2 ± 263.0	1604.0 ± 123.8	2042.0 ± 173.5	<0.001 **	0.888	0.485
Transverse diameter of cardiomyocytes (µm)	19.4 ± 1.3	21.6 ± 1.6	19.4 ± 1.1	19.7 ± 2.0	0.081	0.177	0.184
Thickness of the tunica media (µm)	124.3 ± 6.3	125.1 ± 3.2	117.9 ± 5.1	122.4 ± 11.9	0.446	0.163	0.606
Distance between elastic laminae in the aortic wall (µm)	10.1 ± 1.5	10.6 ± 0.7	9.9 ± 1.5	11.0 ± 1.0	0.212	0.857	0.577
Number of elastic laminae in the aortic wall	10 (9–10)	10 (10–11)	11 (10–11)	11 (10–11)	0.540	0.313	0.540

C: saline solution 0.2 mL/2x/day s.c. for 14 days + saline solution 0.5 mL/day i.p. for 28 days; H: homocysteine 0.45 µmol/g b.w./2x/day s.c. for 14 days + saline solution 0.5 mL/day i.p. for 28 days; C-B6+FA: saline solution 0.2 mL/2x/day s.c. for 14 days + vitamin B6 7 mg/kg b.w. and folic acid 5 mg/kg b.w. i.p. for 28 days; H-B6+FA: homocysteine 0.45 µmol/g b.w./2x/day s.c. for 14 days + vitamin B6 7 mg/kg b.w. and folic acid 5 mg/kg b.w. i.p. for 28 days. n = 10 per group. Data are presented as mean ± SD or median (25th–75th percentile) according to data distribution. * *p* < 0.05; ** *p* < 0.01—two-way ANOVA.

## Data Availability

The original contributions presented in this study are included in the article. Further inquiries can be directed to the corresponding author.

## Abbreviations

The following abbreviations are used in this manuscript:

ANOVA	Analysis of variance
b.w.	Body weight
CAT	Catalase
CBS	Cystathionine β-synthase
CGL	Cystathionine γ-lyase
CSI	Cardio-somatic index
Cys	Cysteine
DNA	Deoxyribonucleic acid
ET-1	Endothelin-1
FA	Folic acid
H&E	Hematoxylin and eosin/phloxine
Hcy	Homocysteine
HHcy	Hyperhomocysteinemia
i.p.	Intraperitoneal
IL	Interleukin
LDH	Lactate dehydrogenase
LDL	Low-density lipoprotein
MDA	Malondialdehyde
MDH	Malate dehydrogenase
Met	Methionine
METC	Mitochondrial electron transport chain
MMP	Matrix metalloproteinase
MS	Methionine synthase
mtDNA	mitochondrial DNA
NADH	Nicotinamide adenine dinucleotide + hydrogen
NF-κB	Nuclear factor kappa B
NO	Nitric oxide
ROS	Reactive oxygen species
s.c.	Subcutaneous
SAAs	Sulfur containing amino acids
SOD	Superoxide dismutase
THF-CH_3_	5-methyltetrahydrofolate
TIMP	Tissue inhibitor of metalloproteinases
TXA_2_	Thromboxane A_2_
U	Units of enzyme activity
